# Role of Dose Intensification for Salvage Radiation Therapy after Radical Prostatectomy

**DOI:** 10.3389/fonc.2016.00048

**Published:** 2016-03-01

**Authors:** Marcus Beck, Tomasz Barelkowski, David Kaul, Sascha Wecker, Alexander H. Thieme, Daniel R. Zwahlen, Peter Wust, Daniel M. Aebersold, Volker Budach, Pirus Ghadjar

**Affiliations:** ^1^Department of Radiation Oncology, Charité Universitätsmedizin Berlin, Berlin, Germany; ^2^Kantonsspital Graubünden, Chur, Switzerland; ^3^Bern University Hospital, University of Bern, Bern, Switzerland

**Keywords:** prostate cancer, salvage, radiation therapy, prostatectomy, dose

## Abstract

For primary radiation therapy (RT) of prostate cancer, dose intensification is established as standard of care. Less is known on the role of dose intensification in the postprostatectomy setting for salvage RT. Thus, we aimed to identify and summarize the existing literature. In retrospective analyses, dose-intensified salvage RT showed a superior biochemical control compared to standard dose salvage radiation with favorable acute and late gastrointestinal and genitourinary toxicity rates, especially when modern radiation techniques such as intensity modulated RT were applied. We identified one randomized phase III trial addressing the potential benefits of dose-intensified salvage RT (SAKK 09**/**10). Recently, acute gastrointestinal and genitourinary toxicities and early quality of life data of this trial were reported, and no significant difference in acute toxicities between both treatment arms were found; however, a significant worsening of genitourinary quality of life was noted in the dose-intensified treatment arm. Whereas dose-intensified salvage RT appears to be feasible and well tolerated, the improved biochemical control rates using dose intensified RT as suggested by retrospective analyses have yet to be validated by prospective trials.

## Introduction

Around 30,000 men will experience recurrence of prostate cancer after radical prostatectomy annually in the United States (1). For the majority of these patients, the only evidence of recurrent disease is an increasing serum PSA level without evidence of macroscopic recurrence. After radical prostatectomy approximately 15–40% of men develop a biochemical relapse within 5 years (2, 3). It has been described that the site of relapse in prostate cancer patients after prostatectomy is predominantly local, with a relatively low incidence of distant failures (4). Patients with biochemical relapse develop bone metastasis with a rate of 37 and 65% at 5 and 10 years, respectively. A median time of 8 years until development of bone metastasis was reported and the observed median time between the development of bone metastasis and death was 5 years (5).

Generally, two main strategies are being used to increase long-term tumor control after prostatectomy: either adjuvant radiation therapy (RT) in the presence of positive surgical margins, extracapsular extension or seminal vesicle invasion, or salvage RT at biochemical relapse. The advantages of dose intensification in primary prostate cancer were already shown in several randomized controlled trials. The meta-analysis by Viani et al. reported a reduction of biochemical relapse rates after dose intensified RT vs. conventional dose RT. However, the dose intensification was associated with increasing rates of grade 2 or higher gastrointestinal toxicity (6).

For the postprostatectomy setting, retrospective analyses have demonstrated the effectiveness of salvage RT in terms of biochemical relapse-free survival and cancer-specific survival (7, 8), and thus salvage RT is considered the only potentially curative treatment at the earliest sign of biochemical failure. Further analyses showed that dose-intensified salvage RT achieved superior biochemical relapse-free survival compared to standard doses (9–11).

As standard for salvage RT, a dose of 64–66 Gy is recommended in the guidelines of the European Association of Urology (EAU) at PSA serum levels of ≤0.5 ng/ml (12). The American Society of Radiation Oncology (ASTRO) guidelines recommend using the highest RT dose deliverable with acceptable toxicity rates and suggest a minimum dose of 64–65 Gy with conventional dose fractionation (13).

Less is known regarding dose intensification in the salvage RT setting. We have thus reviewed and summarized the literature for dose-intensified salvage RT.

## Materials and Methods

Data for this Review were identified by non-systematic searches of MEDLINE, Current Contents, PubMed, and references from relevant articles using medical subject headings including “prostate cancer,” “postoperative,” “radiotherapy,” “radiation,” “adjuvant,” “salvage, dose,” “escalation,” “escalated,” “intensified,” and “intensification.”

## Results

### Retrospective Data

King et al. described improved biochemical relapse-free survival rates for dose-intensified salvage RT (70 Gy) compared to doses of 60 Gy. One hundred twenty-two patients were treated either with a dose of 60 Gy (*n* = 38) or 70 Gy (*n* = 84) using two-dimensional (2D) conformal, three-dimensional (3D) conformal, or intensity modulated RT (IMRT) between 1984 and 2004. Sixty-eight patients received additional androgen deprivation therapy (ADT). The median follow up was >5 years, and patients with 70 Gy treatment had a biochemical relapse-free survival of 58 vs. 25% when treated with 60 Gy. In a multivariate analysis, higher dose was an independent factor for superior biochemical relapse-free survival (9).

Likewise, Ost et al. evaluated 136 patients who received a salvage IMRT with a median dose of 76 Gy alone (*n* = 39) or combined with ADT (*n* = 97) between 1999 and 2008. After a median follow up of 5 years, a biochemical relapse-free survival of 56% and a clinical relapse-free survival of 86% were observed (14). Moreover, Goenka et al. published a retrospective study analyzing 285 salvage RT patients, 72% were treated with a RT dose ≥70 Gy using either 3D or IMRT techniques. Thirty-one percent received additional ADT. The median follow up was 60 months. After 7 years, biochemical relapse-free survival was 37% and distant metastases-free survival was 77% (15). Moreover, a systematic review with regression meta-analysis and radiobiological modeling performed by Ohri et al. analyzed 25 studies with 3,828 patients with a median follow up of 50 month. The RT dose ranged from 60 to 72 Gy (median dose: 65 Gy) and 2D, 3D, or IMRT techniques were applied. The authors observed a median 5-year biochemical relapse-free survival of 47% and detected a dose-related increase of 5-year biochemical relapse-free survival. Each increase of 1 Gy led to an increase of 2.5% of 5-year biochemical relapse-free survival rates (10). Another systematic review published by King analyzed 41 studies with 5,597 patients. The median follow up was 47 ± 22 months, and the applied median dose was 64.6 ± 3.1 Gy. King reported a median relapse-free survival of 34% when RT dose was 60 Gy and 54% with an applied dose of 70 Gy. For each additional 1 Gy, a 2% improvement of relapse-free survival was estimated (16).

The systematic review by Ohri et al. also analyzed the dose-dependent toxicity of 3,828 salvage RT patients treated with 2D/3D or IMRT techniques with a median dose of 65 Gy. A toxicity model was generated and showed increasing dose-dependent rates of ≥grade 3 genitourinary and gastrointestinal toxicity. The authors estimated that with each dose increase of 1 Gy, the rate of ≥grade 3 gastrointestinal late toxicity would increase about 1.2% and grade 3 genitourinary late toxicity rates would increase 0.8%. Furthermore, it was assumed that a rate of >10% late grade 3 gastrointestinal and genitourinary side effects would occur when RT dose exceeds 72 Gy (10). One important limitation of this toxicity model was, however, its dependency on series with 2D/3D treatment techniques. Moreover, the applied doses in the analyzed series were ≤70 Gy. So this model may not be valid to estimate toxicity rates for more modern RT approaches and application of doses >70 Gy (17).

Cozzarini et al. described the long-term toxicity rates of 742 patients treated between 1993 and 2005 with adjuvant RT or salvage RT using 2D and 3D conformal techniques (median follow up 8 years). The salvage RT (*n* = 186) with a median dose of 72 Gy resulted in ≥grade 2 late genitourinary toxicity in 23.7% of the patients. Grade 3 late genitourinary toxicity occurred in 10% of the patients. Grade 2 or higher acute toxicity and a dose of >72 Gy were identified as independent prognostic factors for late grade 3 genitourinary toxicity (18).

Goenka et al. evaluated toxicity rates of 285 patients treated between 1988 and 2007 with salvage RT (median follow up 60 months). One hundred nine patients who received 3D conformal RT (*n* = 12: <66 Gy; *n* = 57: 66 to <70 Gy; *n* = 40: ≥70 Gy) were compared to 176 patients who underwent IMRT (*n* = 3: <66 Gy; *n* = 8: 66 to <70 Gy; *n* = 165: ≥70 Gy). A 8.3% reduction of late ≥grade 2 gastrointestinal toxicity was reported using IMRT (toxicity rate: 1.9%) compared to 3D conformal RT (toxicity rate: 10.2%). In this series, no acute grade 3 gastrointestinal toxicity and only 1.4% late grade 3 gastrointestinal toxicity were observed. The overall ≥grade 2 late genitourinary toxicity rate was 16.3% with no significant difference between the different RT techniques (15, 19). Similar low grade 3 late gastrointestinal toxicity rates were reported by Ost et al. (grade 3 gastrointestinal toxicity <1%) using IMRT (mean dose: 76 Gy) for salvage RT of 136 patients. Late genitourinary ≥grade 2 toxicity rates were 22%, and the grade 3 late genitourinary toxicity rate was 3% (14).

### Prospective Randomized Data

Only one randomized prospective phase III trial testing dose-intensified salvage RT was identified (conducted by the Swiss Group for Clinical Cancer Research, SAKK). The SAKK 09/10 trial was closed for accrual after it met its accrual goal of 350 patients (2011–2014). In this trial, salvage RT with 70 Gy was compared to a dose of 64 Gy. A recent analysis of this trial reported acute toxicity rates and early quality of life in 344 patients being eligible in the safety population (20). European Organization for Research and Treatment of Cancer (EORTC) delineation guidelines were used (21), and toxicity was scored according to National Cancer Institute Common Terminology Criteria for Adverse events (CTC AE, version 4.0). Quality of life was analyzed with the EORTC Quality of Life Questionnaires C30 and PR25. Acute grade 2 genitourinary toxicity occurred in 13%, grade 3 genitourinary toxicity in 0.6% treated with 64 Gy compared to 16.6 and 1.7% grade 2 and 3 genitourinary toxicity after 70 Gy, respectively. Acute grade 2 gastrointestinal toxicity occurred in 16%, grade 3 gastrointestinal toxicity in 0.6% treated with 64 Gy compared to 15.4 and 2.3% acute grade 2 and 3 gastrointestinal toxicity after 70 Gy, respectively. There was no significant difference in acute toxicity rates (CTC AE based) between both arms. Generally, changes in health related quality of life were minor; however, there was a more pronounced and clinically relevant worsening of genitourinary symptoms in the 70 Gy arm. Thus, the initial results of SAKK 09/10 trial confirmed low acute toxicity rates even after dose intensified RT of up to 70 Gy, whereas only slight but significant increase in patient reported early urinary symptoms was shown. In 44% of the patients, the RT was applied using a 3D-conformal approach and in 56% of the patients using an IMRT/rotational RT approach (RT technique was a stratification factor). There was no significant difference in acute gastrointestinal or genitourinary toxicity associated with RT technique (20). The first randomized prospective data regarding freedom from biochemical recurrence (primary trial endpoint) and late toxicity after dose-intensified salvage RT are awaited in 2017.

## Discussion

Retrospective analyses showed improved biochemical control rates after dose-intensified salvage RT with a dose-dependent increase of biochemical relapse-free survival (9–11). The available data suggested slightly increased toxicity rates for dose-intensified salvage RT, when compared to standard dose salvage RT, but both gastrointestinal and genitourinary toxicity rates are generally favorably, even after dose-intensified salvage RT (14, 15, 18, 19).

These findings are confirmed by recent published data from the randomized SAKK 09/10 prospective trial, where low rates of grade 2 and 3 gastrointestinal and genitourinary acute toxicity were described without a significant difference between the two trial arms (total dose 64 vs. 70 Gy). However, there was a significant worsening of genitourinary early quality of life after treatment with 70 Gy. Therefore, some caution might be directed toward impairment of genitourinary early quality of life, which has to be weighed up against potential improvements in biochemical control (20). It was assumed that the necessity to include the bladder neck and the vesico-urethral anastomosis in the high dose salvage RT volume would result in similar genitourinary toxicity regardless of RT technique (17). A worsening of urinary symptoms after high dose RT to urethra, bladder neck, and bladder trigonum was also described in the primary prostate cancer RT (22). This might be the reason for the observed significant worsening of patient who reported urinary symptom burden in the quality of life analysis of SAKK 09/10 (20). It has been well described that patient-reported toxicity scoring systems are more reliable and more sensitive as compared to physician-reported toxicity scoring systems (23), which might be the reason that there was no significant difference in the acute CTC AE-based toxicity scores between the two trial arms.

Interestingly, the SAKK 09/10 trial did stratify for RT technique (3D-conformal RT vs. IMRT/rotational techniques). However, no association was found between RT technique and acute toxicity or early quality of life. This is in contrast to several retrospective analyses that reported that IMRT was associated with a reduced rate of gastrointestinal toxicity as compared to 3D conformal RT in the setting of dose-intensified salvage RT (without a significant difference in genitourinary toxicity) (14, 15, 19). Interestingly, despite being based on retrospective data only, a survey asking physicians in the United States for implemented techniques showed that a majority used IMRT for the salvage RT setting (24). However, in the primary RT setting of prostate cancer (using a higher total dose), preliminary results of the prospective randomized RTOG 0126 trial showed significantly reduced gastrointestinal and genitourinary acute toxicity rates using IMRT compared to 3D conformal RT (25).

In this context, it is important to consider different delineation guidelines with obviously different target volume sizes and its implication for clinical practice. For example, the SAKK 09/10 trial used the EORTC delineation guidelines for clinical (CTV) and planning (PTV) target volumes (20, 21) that were described to be significantly smaller compared to other recommendations such as the Faculty of Radiation Oncology Genito-Urinary Group (FROGG), the Princess Margaret Hospital (PMH), and the RTOG guidelines (26). Hence, the toxicity rates observed in SAKK 09/10 trial and maybe also the differences between the two dose levels in terms of toxicity results could potentially be higher if other delineation guidelines were used.

Otherwise, recently published data confirm that there are other promising treatment options to improve the efficacy of salvage RT. The use of ADT as an additional treatment in the salvage setting was analyzed by two randomized phase III studies. The GETUG-AFU 16 trial compared standard dose salvage RT (66 Gy) alone vs. salvage RT combined with short-term ADT (66 Gy plus 6-month goserelin) and detected a significant improvement in 5-year progression-free survival for the combined treatment. More acute toxicities (<grade 3) were observed after combined treatment but acute grade 3 or late toxicity was not significantly different between the trial arms (27). Moreover, the long-term results of RTOG 9601, a trial that compared standard dose salvage RT (64.8 Gy) vs. salvage RT with long-term ADT (64.8 Gy plus 24-month bicalutamide), showed a significant overall survival benefit after 10 years with 78% for RT alone vs. 82% for the combined treatment (hazard ratio 0.75, 95% CI: 0.58–0.98). No significant difference in grade 3 or 4 late toxicity was described, whereas significantly more gynecomastia was observed in the bicalutamide group (70%) vs. RT (11%) (28). However, both studies applied a standard dose RT, and thus no firm conclusions about combination of ADT with dose-intensified salvage RT can be made and whether this would lead to a similar or better outcome or to unacceptable toxicity. Thus, it has to be considered that ADT is associated with multiple short and long-term side effects like bone loss, sexual dysfunction, hot flashes, metabolic changes, fatigue, gynecomastia among others (29). It might be that dose-intensified salvage RT alone achieves similar results without ADT-associated side effects or at least might be capable to significantly delay the use of ADT. Another potential option to achieve improved outcome in the salvage RT is regional hyperthermia, which will be investigated in a novel phase II trial (30).

Finally, until biochemical relapse-free survival and late toxicity rates from the SAKK 09/10 trial become available, the described worsening of genitourinary quality of life after 70 Gy must be weighted up against benefits in cancer control, potentially being obtained by dose intensification. For patients without macroscopic recurrence, one practical solution could be to deliver dose-intensified salvage RT up to 70–72 Gy in the absence of acute genitourinary toxicity but to stop the salvage RT after 66 Gy in the presence of significant acute toxicity. Alternatively, a simultaneous integrated boost technique (SIB) might be applied to selectively apply a higher dose to the high-risk quadrant of the prostatic bed (e.g., pT3, R1) while sparing the vesico-urethral anastomosis/urethra if possible. Patients with macroscopic recurrences will probably benefit from higher doses (toward 76 Gy) and the addition of ADT.

## Conclusion

According to retrospective data, dose-intensified salvage RT appears to be well tolerated and effective. However, a slight increase in acute and late toxicities using dose-intensified salvage radiation treatment could be detected. A prospective trial reported favorable acute toxicity rates after dose-intensified salvage RT, but biochemical control rates and late toxicity data of this trial are still pending. As long as these prospective data are not available, the potential benefits in biochemical control and the mild increase of toxicities in dose-intensified salvage RT (both reported in retrospective studies) have to be weighed up.

## Author Contributions

MB and PG participated in drafting and revising the manuscript; TB, DK, SW, AT, DZ, PW, DA, and VB participated in revising the manuscript.

## Conflict of Interest Statement

The authors declare that the research was conducted in the absence of any commercial or financial relationships that could be construed as a potential conflict of interest.
